# Associations of Sleep Quality and Apolipoprotein Genotype with Blood‐based Biomarkers of Alzheimer's Disease and Neurodegeneration

**DOI:** 10.1002/alz70856_099180

**Published:** 2025-12-24

**Authors:** Tina T. Vo, Christine Fennema‐Notestine, Carol E. Franz, Nathan A. Gillespie, William S. Kremen, Michael J. Lyons, Chandra A. Reynolds, Robert A. Rissman, Jeremy A. Elman, Matthew S. Panizzon

**Affiliations:** ^1^ University of California San Diego, La Jolla, CA, USA; ^2^ Center for Behavior Genetics of Aging, University of California, San Diego, La Jolla, CA, USA; ^3^ Virginia Commonwealth Univeristy, Richmond, VA, USA; ^4^ Boston University, Boston, MA, USA; ^5^ University of Colorado Boulder, Boulder, CO, USA; ^6^ Institute for Behavioral Genetics, University of Colorado Boulder, Boulder, CO, USA; ^7^ Alzheimer's Therapeutic Research Institute, Keck School of Medicine, University of Southern California, San Diego, CA, USA; ^8^ University of California, San Diego, La Jolla, CA, USA

## Abstract

**Background:**

Poor sleep quality (SQ) is commonly observed in individuals with Alzheimer's disease and related dementias (AD/ADRD), with emerging evidence suggesting it may increase risk, albeit with unclear mechanisms. As such, the present study examined whether SQ and its interaction with Apolipoprotein ɛ4 (*APOE* ɛ4) are associated with blood‐based biomarkers, particularly AD‐related markers of β‐amyloid, tau, neurodegeneration, and inflammation.

**Method:**

Leveraging cross‐sectional data from the Vietnam Era Twin Study of Aging (VETSA; *N* = 938, *M*_age_=67.39 [range=61.37‐73.25]), we examined these associations in a large sample of early old‐age men. Measures included self‐reported SQ (Pittsburgh Sleep Quality Index total score, higher scores indicate poorer SQ) and a weighted *APOE* score based on an individual's number of ɛ2 and ɛ4 alleles, with higher risk (i.e., ɛ4 alleles) assigned larger weights. Blood‐based biomarkers included β‐amyloid 42 to β‐amyloid 40 ratio (Aβ42/40), phosphorylated tau (pTau231), neurofilament light chain (NFL), and glial fibrillary acidic protein (GFAP). All models were adjusted for age and health status.

**Result:**

Poorer SQ was significantly associated with elevated pTau231 (β = 0.02, *p* = 0.04), which may reflect greater disease‐related accumulation. The weighted *APOE* score was associated with lower Aβ42/40 ratio (β=‐0.25, *p* < .001), and increased levels of GFAP (β=0.17, *p=*0.005), suggesting that higher genetic risk for AD was associated with greater levels of AD‐related pathology and inflammation. Notably, while the weighted *APOE* score alone was not significantly associated with pTau231 levels, its interaction with SQ was significant such that the association between SQ and pTau231 was weaker in individuals with higher genetic risk (β=‐0.04, *p=*0.01).

**Conclusion:**

As elevated pTau231 levels may be associated with the development and progression of AD, poor SQ may also be linked to early stages of these processes. These results cannot address direction of causation, but for individuals at lower genetic risk for AD based on *APOE*, poorer SQ might represent an alternative pathway influencing AD pathogenesis. If so, these findings underscore the importance of addressing SQ as a modifiable risk factor that may reduce the risk of AD and they highlight the need to consider genetic risk when examining SQ.

